# Differential expression of ANXA1 in benign human gastrointestinal tissues and cancers

**DOI:** 10.1186/1471-2407-14-520

**Published:** 2014-07-19

**Authors:** Yunshu Gao, Ying Chen, Dongyun Xu, Jiejun Wang, Guanzhen Yu

**Affiliations:** 1 Department of Medical Oncology, Changzheng Hospital, Shanghai, China; 2 Department of Oncology, 401 Hospital of PLA, Qingdao, Shandong Province, China; 3 Department of Pathology, Changhai Hospital, Shanghai, China; 4 Department of Oncology, the 97 Hospital of PLA, Xuzhou, Jiangsu Province, China

## Abstract

**Background:**

Annexin-1 contributes to the pathological consequence and sequelae of most serious human diseases including cardiovascular disease and cancer. Although diverse roles in carcinogenesis have been postulated, its role in human gastrointestinal cancers still remains controversial.

**Methods:**

The mRNA and protein expression profiles of ANXA1 were studied in human esophageal, gastric, pancreatic, colorectal, liver, and bile duct cancers using Real-Time PCR, western blotting, and immunohistochemistry. Gain/loss-of-function by pcDNA3.1-ANXA1 and ANXA1-shRNA was performed in gastric cancer cells.

**Results:**

ANXA1 was widely expressed in adult gastrointestinal tissue. All methods showed that ANXA1 was down-regulated in esophageal, gastric, and bile duct cancers, but up-regulated in pancreatic cancer. Forced ANXA1 expression in gastric cancer cells leads to cell growth inhibition and concomitantly modulates COX-2 expression. We confirm loss of ANXA1 and overexpression of COX-2 in clinical gastric cancer, suggesting that the anti-proliferative function of ANXA1 against COX-2 production might be lost.

**Conclusions:**

ANXA1 expression is “tumor-specific” and might play a multifaceted role in cancer development and progression. ANXA1 was widely expressed in normal gastrointestinal epithelium, suggesting its role in the maintenance of cellular boundaries. Furthermore, ANXA1 regulates GC cell viability via the COX-2 pathway.

## Background

The annexin superfamily consists of 13 calcium or calcium and phospholipid binding proteins expressed in most eukaryotic cell types. Despite their high biological and structural homology (40-60%), annexins have diverse functions in cellular activities including vesicle trafficking, cell division, apoptosis, calcium signaling, and growth regulation. In certain clinical conditions, the expression levels of annexins or their localization changed remarkably, which suggests that annexins may contribute to the pathological consequence and sequelae of most serious human diseases including cardiovascular disease and cancer [1]. As the first characterized member of the annexin superfamily, Annexin-1(ANXA1) gene located on chromosome 19q24, encoding a 37 kDa protein functioning as a strong inhibitor of glucocorticoid-induced eicosanoid synthesis and PLA2. Recently, increasing evidences implicated that ANXA1 contributes to a variety of cellular biological activities, including anti-inflammatory effects, cell proliferation inhibition, the regulation of cell death and differentiation, phagocytic clearance of apoptosing cells, and most importantly the process of carcinogenesis [2].

These diverse biological activities of ANXA1 make it a potential target for novel therapeutic intervention. However, more recently, ANXA1 protein has been recognized to be differentially expressed in various human tumors, e.g., breast cancer, prostate cancer, esophageal cancer, gastric cancer, endometrial carcinoma, pancreatic cancer, and colorectal cancer [2-13]. Loss/aberrant expression pattern of ANXA1 in esophageal squamous cell carcinoma, prostate cancer, and endometrial carcinoma could be correlated with altered tumor behavior, e.g., dedifferentiation of tumor cells, increased invasiveness, and thus with tumor progression [6,8-10,12]. On the other hand, increased expression pattern of ANXA1 in hepatocellular carcinoma, colorectal cancer, and pancreatic cancer was shown to be associated with tumor growth, lymph node metastasis, and advanced disease stages, and consequently with poor patient outcome [11,13,14]. However, contradictory descriptions on ANXA1 expression were reported in certain human tumors, e.g., breast cancer [3,5,15], bladder cancer [16,17], and gastric cancer as well [18-21]. Hence, although the importance of ANXA1 in cancer is apparent and antibodies for therapeutic invention were easily prepared, researchers and clinicians are hampered by the conflicting expression pattern of ANXA1 in human solid cancers and by the lack of complete data sets describing the tissue-specific expression of this gene/protein.

We therefore sought to systematically investigate the expression pattern of ANXA1 in human gastrointestinal solid cancer and matched non-cancerous tissues. Generally, real-time RT-PCR was used for the detection of ANXA1 mRNA expression, while Western blotting and immunohistochemistry were established to visualize the tissue-specific expression pattern and quantification of ANXA1 protein in these specimens. Next, we restored ANXA1 expression in AGS gastric cell lines and the possible mechanism of ANXA1 in gastric carcinogenesis was further explored. These differentially expression patterns of ANXA1 in gastrointestinal carcinomas set a solid groundwork for further ANXA1-targeted molecular cancer therapy and as a diagnostic and prognostic marker.

## Methods

### Isolation of RNA

The RNA was collected from 6 different human gastrointestinal tumor entities and matched non-cancerous human tissues. RNA was pooled from each tissue to equalize potential interindividual differences. The detailed sample information can be obtained from Table 1. Human tissue RNA was obtained from the Department of Pathology, Changhai Hospital except for cholangiocarcinoma and matched non-cancerous tissue RNA. The RNA of four additional bile ducts and cholangiocarcinoma was harvested from specimens resected in the Department of Surgery, Eastern Hepatobiliary Hospital. Gastric cell lines (GES1, SGC7901, HGC27, MKN45, MGC803, N87, AGS and BGC823) were purchased from the Cell Center of Chinese Academy of Sciences, Shanghai, China.

**Table 1:** Detailed information of the samples obtained from gastrointestinal tract

**No.**	**Tissue**	**Patients, N**	**Gender**	**Ethnic group**	**Histology**
1	Esopheagus	4	M/F	Asian	Normal
2	Stomach	8	M/F	Asian	Normal
3	Pancreas	4	M/F	Asian	Normal
4	Colon and rectum	4	M/F	Asian	Normal
5	Liver	10	M/F	Asian	Normal
6	Bile duct	4	M/F	Asian	Normal
7	Esopheageal cancer	4	M/F	Asian	Squamous cell carcinoma
8	Gastric cancer	8	M/F	Asian	Adenocarcinoma
9	Pancreatic cancer	4	M/F	Asian	Ductal adenocarcinoma
10	Colorectal cancer	8	M/F	Asian	Adenocarcinoma
11	Liver cancer	10	M/F	Asian	Hepatocellular carcinoma
12	Cholangiocarcinoma	4	M/F	Asian	Adenocarcinoma

Briefly, one part of the specimens from non-cancerous tissues and tumors were submersed in liquid nitrogen immediately after resection. Additional parts of the tissue samples were used for H&E staining to control the percentage of tumor cells. After disruption with a mortar and pestle, samples were shredded in a Qiagen shredder column for homogenization. RNA isolation was carried out according to the protocol of the RNeasy Mini Kit (Qiagen) and RNA quality and quantity were assessed using the Agilent 2100 bioanalyzer (Agilent Technologies). The RNA was split into several parts and stored at -80°C.

### Real-time RT-PCR

First-strand cDNA was synthesized with the Reverse Transcription Kit from Promega (Madison) according to the manufacturer’s protocol. SYBR Green) real-time RT-PCR was performed on LC480II Sequence Detection System (Roche) to quantify the transcribed gene-specific RNA. Primer sequences are as follows: ANXA1: forward, 5- GCAGGAATATGTTCAAACTGTG-3 and reverse, 5- CCTTATGCAAGGCAGCGA-3; GAPDH: forward, 5- TGTTGCCATCAATGACCCCTT-3 and reverse, 5- CTCCACGACGTACTCAGCG-3. Standard curve method was used to determine the relative amount o ANXA1 expression. Of the 6 non-cancerous tissues, the one with lowest expression of ANXA1 was set as the calibrator, while GES1 was set as the calibrator of the 8 gastric cell lines. The other normalized value was divided by the calibrator representing the x-fold mRNA amount compared to this calibrator in figures [22]. Of the cancerous tissues, the normalized value was calculated as C = ㏒ mRNA amount in tumor tissues/mRNA amount in matched adjacent non-cancerous tissues.

#### ***Western blotting analysis***

Standard Western blotting was done as previously described [21]. Briefly, after homogenization of the samples described above, lysis buffer (50 mM Tris–HCl [pH7.6], 150 mM NaCl, 1% Nonidet P-40, 0.5% sodium deoxycholate, 0.1% SDS) with a protease inhibitor cocktail (Nacalai Tesque, Kyoto, Japan). The whole tissue/cell lysates were incubated on ice for 15 min and then centrifuged at 14,000 rpm for 30 min at 4°C. A Bradford ULTRA Total Protein Quantitation Kit (Novexin Ltd., Cambridge, UK) was performed to detect the protein concentrations of the supernatants. Equal amount of lysates were resolved by SDS/PAGE and transferred electrophoretically to PVDF membrane (Bio-Rad). The membranes were probed with specific antibodies and the immunoreactive proteins were detected by the enhanced chemiluminescene (ECL) kit (Santa Cruz Biotechnology). Rabbit anti-Annexin A1 (1:1,000; ab33061) was purchased from Abcam Company (Cambridge, UK). Antibodies to COX-2 (D5H5) and cyclin D1 (92G2) were purchased from Cell Signaling Technology (Beverly, MA) and β-actin antibody were from Santa Cruz Biotechnology (Santa Cruz, CA).

#### ***Immunohistochemistry***

For the immunohistochemical analysis of ANXA1 protein expression in gastrointestinal cancers, paraffin embedded tissue specimen of 19 esophageal, 52 gastric, 32 colorectal, 47 pancreatic, and 8 hepatocellular carcinoma and matched adjacent normal tissues were retrieved from the Department of Pathology, Changhai Hospital. 20 hilar cholangiocarcinomas were obtained from the Department of pathology, Eastern Hepatobiliary Hospital. All patient samples (RNA and paraffin embedded tissue specimens) recruited to our study were approved by the ethical review committees (Institutional Review Board of Eastern Hepatobiliary Hospital, Shanghai and Institutional Review Board of Changzheng Hospital, Shanghai). Consecutive sections (4 μm) of paraffin-embedded normal and tumor specimens were prepared and processed for immunohistochemical analysis as described previously. ANXA1 protein expression in these sections and COX-2 in gastric cancers were detected with appropriate antibodies against ANXA1 (1:100) and COX-2 (1:150). Two individuals (G.Y. and Y. C.) scored these sections without the knowledge of patients’ characteristics using an Olympus CX31 microscope (Olympus Optical). According to the staining intensity, these proteins’ staining was classified as follows: 0 for absence of staining, 1 representing mild expression, 2 representing moderate expression, or 3 representing high expression. A mean percentage of positive tumour cells were determined in at least five areas at 400 magnifications and assigned from 0 to 100. The percentage of positive tumour cells and the staining intensity were multiplied to produce a weighted score for each case. Theoretically, the scores ranged from 0 (0% of cells staining) to 3 (100 × 3/100).

#### ***Immunofluoresncece analysis***

The sections of the paraffin embedded tissues were deparaffinized and rehydrated. Gastric cancer cells (N87 and AGS) were fixed with 4% formaldehyde in PBS for 15 min and rinsed 3 times in PBS for 5 min. Gastric tissues and cells were blocked with blocking buffer (4% Goat serum) for 60 min and then applying diluted anti-ANXA1 antibody (1:100) overnight at 4°C. Then the specimens were incubated with fluorochrome-conjugated secondary antibody for 1h. After PBS washing, nuclei were stained with DAPI (Sigma, Munich, Germany) and examined by fluorescence microscopy (Keyence, Neu-Isenburg, Germany).

#### ***Cell proliferation assay***

pcDNA3.1 vector and pcDNA3.1-ANXA1 plasmids were kind gifts from Prof. Minghua Zhu (Department of Pathology, Changhai Hospital, Shanghai, China). GV147-COX2 plasmid was purchased from Genechem Company, Shanghai, China. The shRNA-ANXA1 and unspecific scrambled shRNA plasmids were purchased from Origene Technologies, Maryland, US. At 24 hours before transfection, 1 × 10^5^ cells were seeded in six well plates. Transfection of the above plasmids was carried out using 15 ul Lipofectamine™ 2000 reagent (Invitrogen, Karlsruhe, Germany) and 5 ng plasmid per well according to the manufacturer’s instructions.

At 12 hours after transfection, cells were digested and 5000 cells were seeded in 96-well plates and incubated in medium with 10% FBS. At 24 h, 48 h, and 72 h, CCK8 assay (Dojindo Kumamoto, Japan) was performed to measure the final results. The experiment was repeated three times independently.

#### ***Colony formation assay***

At 24 hours after transfection, cells were digested and seeded in 6-well plates in triplicate at a density of 500 cells/well for 14 days at 37°C. The colonies were fixed with methanol/acetone (1:1) and stained with crystal violet. Colonies with cell numbers of more than 50 cells per colony were counted.

#### ***Transwell migration assay***

The migration assay was performed in a 24-well transwell cell culture apparatus with multiporous polycarbonate membrane insert (8-μm pore size) (Corning). Briefly, cells were collected and resuspended in serum-free media at a density of 1 × 10^5^ cells/ml. The top chamber was loaded with 100 μl cell suspension and the lower chamber was filled with 500 μl media with 10% FBS. After incubation at 37°C in 5% CO2 for 24 h, the filters were removed, rinsed 3 times with PBS, fixed with methanol, and stained with 0.5% crystal violet reagent. Migrated cells were determined by counting specified cross-sectional fields on the lower side of filters with a phase-contrast micro-scope.

#### ***Statistical analysis***

Categorical data were analyzed using x^2^ tests. The significance of the in vitro data was determined using two-tailed Student’s t test or Two-way ANOVA. Within-group correlations of continuous and ordinal variables were assessed using Pearson’s correlation coefficient or T test when appropriate. Analyses were done using the SPSS statistical software program for Microsoft Windows (SPSS). In all of the tests, a two-sided P < 0.05 was defined as statistically significant.

## Results

### ANXA1 expression profile in non-cancerous human gastrointestinal tissues

**Figure 1:**
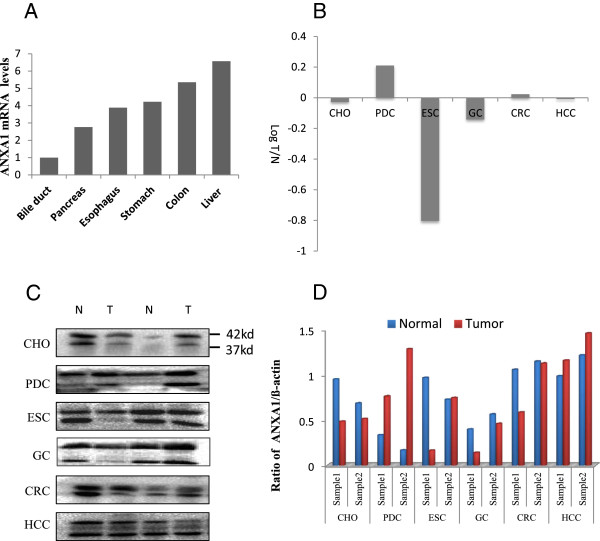
**Expression profiles of ANXA1 in human gastrointestinal carcinomas and noncancerous tissues by Real-time PCR and Western blot analysis. (A)** Normalized mRNA values of ANXA1 in human gastrointestinal tissues. Bile duct tissue with the lowest expression of ANXA1 mRNA was designated as the calibrator and the normalized value for each tissue was divided by this calibrator. **(B)** Differential expression of ANXA1 mRNA in 6 different tumor types. The fold changes (*y axis*) are calculated by the ratio of the relative amounts of mRNA in the tumor *vs* healthy tissue in log scale. **(C)** Differential expression of ANXA1 protein in the six tumor types determined by western blotting assay. N, matched noncancerous tissue; T, tumor. ANXA1: 37kd, ß-actin: 42kd. **(D)** Graphical representation of the ratio of T/N in these six types of tumor from **(C)**. N, matched noncancerous tissue; T, tumor. CHO, cholangiocarcinoma; PDC, pancreatic carcinoma; ESC, esophageal carcinoma; GC, gastric carcinoma; CRC, colorectal carcinoma; HCC, hepatocellular carcinoma.

**Figure 2:**
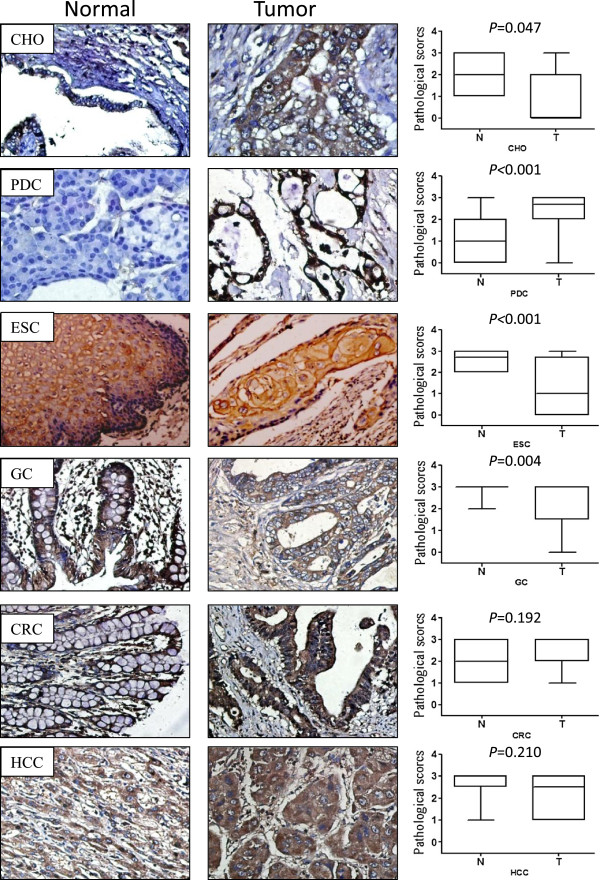
**Immunohistochemical staining for ANXA1 protein in the six gastrointestinal tumors (middle) and matched noncancerous tissues (left) and Graphical representation of the intensity of ANXA1 staining in the specimens (right).** IHC × 200. T tests were used to analyze the differences of ANXA1 protein level between primary tumor and matched non-cancerous tissues from gastrointestinal tract.

The quantitative ANXA1 mRNA expression profile was investigated in normal human gastrointestinal tissues using real-time RT-PCR. Interestingly, ANXA1 was expressed in all investigated healthy tissues, but the relative amounts of ANXA1 transcript varied considerably. Bile duct tissue displayed the lowest expression of ANXA1 and is designated as the calibrator (Figure 1A). In contrast, liver tissue displays the highest relative mRNA expression compared to the other 5 tissues. ANXA1 was also expressed at relatively high levels in the esophagus, stomach, and colon tissues. Pancreas was the organ with the second lowest ANXA1 mRNA expression after bile duct. Western blotting results revealed high level of ANXA1 protein expression in gastrointestinal healthy tissues except pancreas (Figure 1C, low band). Weak ANXA1 expression could be detected in pancreas. Similar results were found using immunohistochemical analysis. ANXA1 was preferentially highly expressed in liver, esophagus, stomach, and colon tissues, but low in pancreas (Figure 2, left). Interestingly, in contrast to lowest level of ANXA1 mRNA in bile duct, ANXA1 protein was moderately expressed in the epithelium of bile duct (Figure 1C).

#### ***ANXA1 expression profile in human gastrointestinal carcinomas***

Since ANXA1 has been proved to contribute to the tumorigenesis of various human malignancies, we investigated six of the more frequently occurring tumor entities in human gastrointestinal system (esophagus, stomach, colon, liver, bile duct, and pancreas) for ANXA1 expression to reveal its potential role as a diagnostic marker or as a therapeutic target. In the esophageal squamous cell carcinomas, ANXA1 mRNA expression was 6-fold lower than in the noncancerous colon tissue (Figure 1B). Similarly, ANXA1 protein expression was also down-regulated in squamous cell carcinoma than in surrounding normal esophageal epithelium (Figure 1C, D and Figure 2 third panel). Immunohistochemistry for ANXA1 revealed high expression level of this protein in well-differentiated squamous cell carcinomas, but weak or not in poorly differentiated carcinomas (Figure 2 third panel) (Additional file 1: Figure S1). In contrast to esophageal carcinoma, ANXA1 mRNA was up-regulated 1.6-fold in pancreatic carcinomas (Figure 1B). Furthermore, investigation of ANXA1 protein level revealed extremely stronger expression of this protein in pancreatic carcinomas than in the surrounding benign pancreatic tissue (Figures 1C, D and 2 second panel). However, ANXA1 expression, either at mRNA level or at protein level, was not changed significantly in colorectal and hepatocellular carcinomas compared with that in the surrounding benign tissues (Figures 1C, D and 2 fifth to sixth panel). In hilar cholangiocarcinoma, ANXA1 mRNA changed a little, but ANXA1 protein was sharply down-regulated compared with surrounding noncancerous tissue revealed by immunohistochemical analysis (Figures 1C, D and 2 first panel). Similar to cholangiocarcinoma, in gastric carcinomas, ANXA1 mRNA was slightly down-regulated 1.2-fold (Figure 1B). However, ANXA1 protein expression was almost completely lost in a number of gastric cancer specimens (Figures 1C, D and 2 forth panel). Immunohistochemistry for ANXA1 in cholangiocarcinoma and gastric carcinoma displayed positively staining cells mainly localized in well-differentiated carcinomas and rarely in poorly differentiated carcinomas.

#### ***ANXA1 expression profile in human gastric cancer***

**Figure 3:**
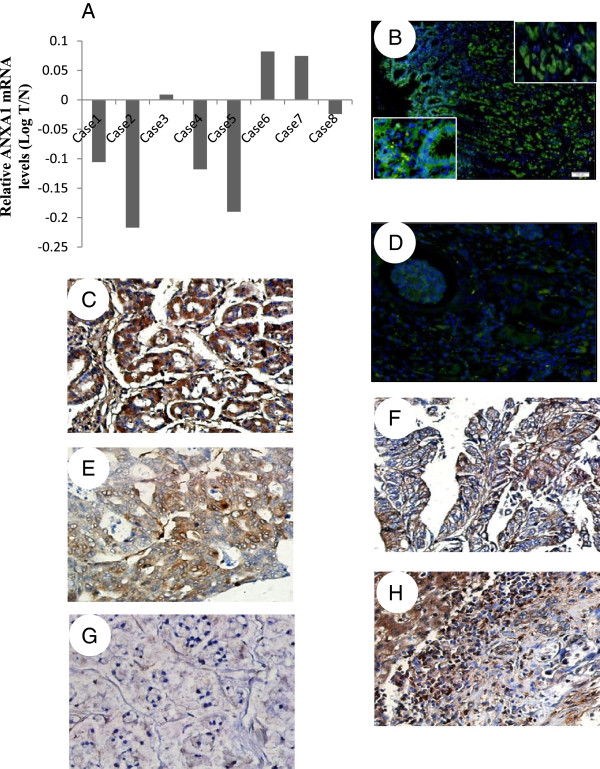
**Expression profile of ANXA1 in human gastric carcinomas and noncancerous tissues. (A)** Level of ANXA1 mRNA in 8 gastric cancer cases. The fold changes (*y axis*) are calculated by the ratio of the relative amounts of mRNA in the tumor *vs.* healthy tissue in log scale; **(B)** ANXA1 expression in normal gastric mucosa by immunofluoresncece assay. **(C)** ANXA1 expression in normal gastric mucosa by immunohistochemistry. **(D)** ANXA1 expression in gastric cancer specimens by immunofluoresncece assay. **(E-F)** ANXA1 expression in gastric cancer specimens by immunohistochemistry. **(E)** Negative ANXA1 expression in intestinal types; **(F)** Positive ANXA1 expression in intestinal types; **(G)** Negative ANXA1 expression in diffuse types; **(H)** High level of ANXA1 expression in adjacent liver tissues and low level of ANXA1 expression in metastatic tumor cells. Original magnification: 400×.

**Figure 4:**
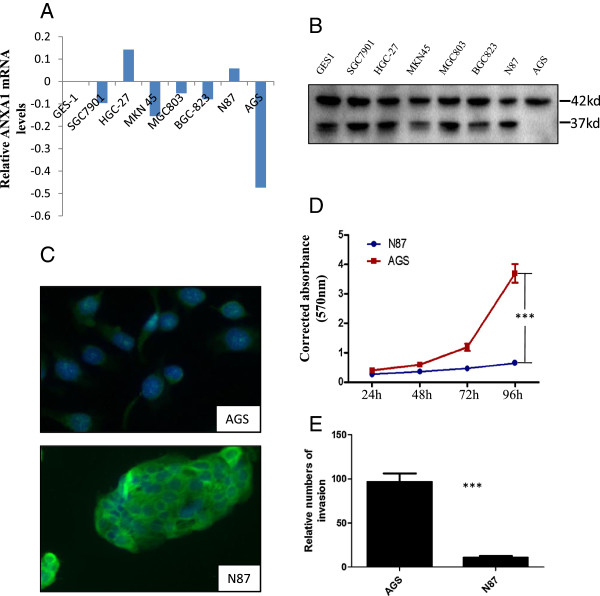
**Expression of ANXA1 in human gastric cancer cells. (A, B)** Levels of ANXA1 mRNA **(A)** and protein **(B)** in 7 gastric cancer cell lines and 1 immortalizated gastric cell, GES1; ANXA1: 37kd, ß-actin: 42kd. **(C)** Localization and intensity of ANXA1 in AGS and N87 cell lines determined by immunofluorescence analysis; **(D)** Proliferative rates of AGS and N87 determined by CCK8 assay; Two-way ANOVA was performed to analyze the overall difference of the proliferation rate between two cell types. ***P < 0.001. **(E)** Differences of invasion ability of AGS and N87 shown by two-chamber invasion assay. Unpaired t test was carried out to analyze the difference of the invasion cell numbers between the two cell types. ***P <0.001.

Since investigation of the pooled RNA of eight gastric cancer specimens revealed a slight decrease of ANXA1 compared with noncancerous gastric mucosa, we then detected the quantitative expression of ANXA1 in these eight cases, respectively, and in eight gastric cells (1 immortalizated gastric cell, GES1, and 7 gastric cancer cell lines). Five of these cases showed a decreased expression of ANXA1 in gastric carcinomas compared with surrounding normal gastric tissue, while the other three showed an increased expression of ANXA1 (Figure 3A). Furthermore, histological analysis revealed that tumors with ANXA1 up-regulation tend to be well-differentiated or at early disease stage, while those with ANXA1 down-regulation tend to be poorly differentiated. To confirm our findings, we investigated ANXA1 expression in a cohort of 52 gastric cancer cases and compared the results with expression in surrounding benign tissue. As shown in Figure 3B, C and Additional file 2: Figure S2, ANXA1 was profoundly expressed both in gastric mucosa and in gastric glands of all gastric tissues. In tumor, however, loss of ANXA1 expression was observed in 31 (59.6%) of the 52 primary gastric tumors. Of 21 ANXA1-positive cases, ANXA1 expression was significantly associated with histological differentiation: 100% (3/3) in well-differentiated tumors, 51.9% (14/27) in moderately differentiated tumors, and 18.2% (4/22) in poorly differentiated tumors (*P* = 0.005) (Figure 3E-G). Specifically, immunohistochemistry for ANXA1 in liver metastases displayed a significant reduction of this protein in tumor cells (Figure 3H). Therefore, ANXA1 mRNA and protein expression in gastric cancer seemed to be fairly congruent: ANXA1 is a differentiation marker.Immortalizated gastric cell, GES1, served as the calibrator and ANXA1 mRNA was down-regulated in 5 of 7 gastric cancer cell lines, whereas up-regulated in HGC-27 and N87 cell lines (Figure 4AF). ANXA1 protein levels were almost in line with ANXA1 mRNA levels in these cells (Figure 4B). Immunofluorescence analysis confirmed high expression of ANXA1 in N87 cell lines and low in AGS cell lines (Figure 4C). Interestingly, AGS cells have higher ability of proliferation and migration than N87 cells (Figure 4D, E). Thus, ANXA1 expression levels correlated closely with cell growth and migration in these cell lines.

### ANXA1 inhibits proliferation and migration of human GC cells

**Figure 5:**
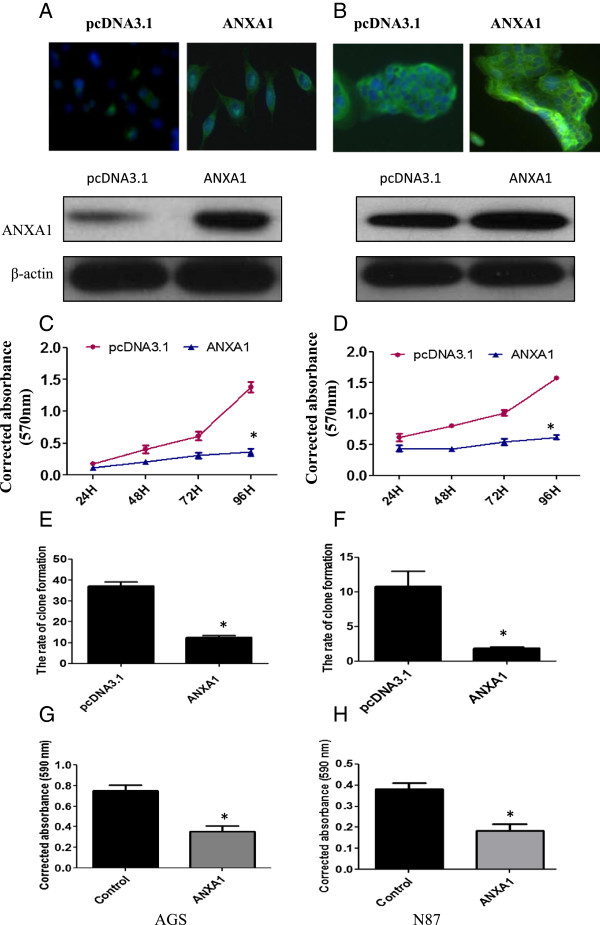
**ANXA1 regulates cell viability and invasion of gastric cancer cells. (A, B)** Immunofluorescence analysis (upper panel) and western blot analysis (lower panel) showed ANXA1 expression from AGS (left) and N87 (right) cancer cells that were transfected with pcDNA3.1-ANXA1 expression vectors. **(C-H)** Gastric cancer cells (AGS and N87) were transfected with pcDNA3.1 vector or pcDNA3.1-ANXA1 expression vectors and cell proliferation **(C, D)**, colony formation **(E, F)** and invasion ability **(G, H)** were performed. The histogram shows the percentage of cells that formed colony or penetrated the membrane. Two-way ANOVA was performed to analyze the overall difference of the proliferation rate between cells transfected with pcDNA3.1 vector or pcDNA3.1-ANXA1. *P < 0.05 as compared to the control. Unpaired t test was carried out to analyze the difference of the ability of colony and invasion between the two groups. *P < 0.05 as compared to the control.

**Figure 6:**
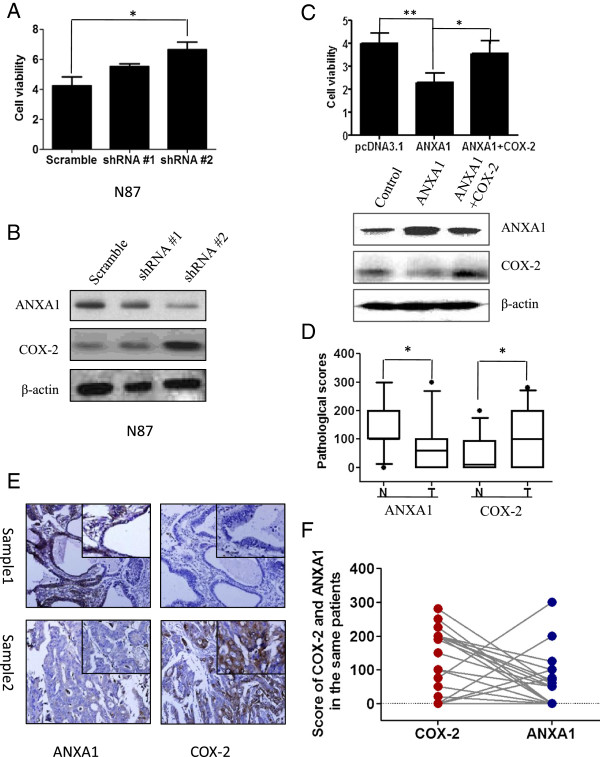
**ANXA1 expression inversely significantly correlated with COX-2 expression. (A)** CCK8 assay showed that ANXA1 down-regulation leaded to increased cell viability of N87. The results are expressed as the ratio of viable cells at 48 h compared to that at 0 h. *P < 0.05 as compared to the control. **(B)** Western blot analysis showed ANXA1 and COX-2 expression in N87 cancer cells with ANXA1 down-regulation. **(C)** Upper: CCK8 assay showed that overexpression both ANXA1 and COX-2 simultaneously abolish the effect of inhibition of cell proliferation by ANXA1 alone in AGS cells. Lower: Western blot analysis showed ANXA1 and COX-2 expression in AGS cancer cells with ANXA1 overexpression alone or both ANXA1 and COX-2 overexpression. **(D)** Graphical representation of the intensity of ANXA1 and COX-2 staining in gastric cancer and non-cancerous tissues. N, non-cancerous tissues; T: primary tumor tissues. T tests were used to analyze the difference of the proliferation rate from each group or the intensity of ANXA1 and COX-2 protein levels between gastric cancer and non-cancerous tissues. *P < 0.05 as compared to the control. **(E)** Representative pictures showing expression patterns of ANXA1 and COX-2 in human gastric cancers from the same patients. Original magnification of the big pictures: 100×; Original magnification of the small pictures: 400×. **(F)** Graphical representation of levels of ANXA1 and COX-2 immunostaining in human gastric cancers from the same patients.

Next, to investigate whether GC cell proliferation and migration can be affected by ANXA1, AGS and N87 cells were treated with either pcDNA3.1 vector or full-length ANXA1/pcDNA3.1. Levels of ANXA1 expression was increased in AGS and N87 cells induced ANXA1/pcDNA3.1 transfection (Figure 5A, B). Proliferation analysis showed that full-length ANXA1-transfection leads to proliferation arrest of AGS and N87 cancer cells (Figure 5C, D). Exogenous overexpression of ANXA1 significantly impaired the colony formation and migration of AGS and N87 cancer cells (Figure 5E-H). However, silencing of ANXA1 by ANXA1-shRNA promoted cell viability in N87 cells (Figure 6A).

### ANXA1 abrogates COX-2 expression in GC cells

Since ANXA1 has anti-inflammatory effects and COX-2 is an important mediator in inflammation, we investigated the expression of COX-2 in GC cells when transfected with pcDNA3.1-ANXA1 or ANXA-shRNA. Western blotting analysis revealed that silencing of ANXA1 leaded to up-regulation of COX-2 (Figure 6B), while forced expression of ANXA1 decreased COX-2 production (Figure 6C). In addition, overexpression both ANXA1 and COX-2 simultaneously abolish the effect of inhibition of cell proliferation by ANXA1 alone (Figure 6C).

Furthermore, we examined COX-2 expression in these gastric cancer cases and investigated their correlation with ANXA1 expression. Immunohistochemical analysis revealed a higher expression of COX-2 and a lower expression ANXA1 in gastric tumors than those in non-cancerous tissues (Figure 6D). In addition, ANXA1 positive expression negatively correlated significantly with COX2 (r = -0.500, *P* = 0.011) (Figure 6E and F). These data suggested tumor suppressor function of ANXA1 to inhibit proliferation partly through regulating the production of COX-2.

## Discussion

In the present study, a systematic expression profile of ANXA1 in human gastrointestinal tumors was explored by real-time RT-PCR, Western blot, immunohistochemistry, and/or immunofluoresncece. We provided comprehensive evidences that (1) ANXA1 is differentially expressed in healthy human tissues; (2) ANXA1 expression is tumor type-specific: downregulation in squamous cell carcinoma, upregulation in pancreatic carcinoma, but controversial in other gastrointestinal tumors; (3) enforced expression of ANXA1 reduced cell viability by inhibiting the production of COX-2, while COX-2 overexpression could abolish this effect induced by ANXA1. Our findings can be helpful in disclosing the functional diversity of ANXA1 on the basis of organ-specific expression profiles.

In contrast to various studies that various gastrointestinal epithelial tissues showed weak or absent expression of ANXA1, our findings revealed that ANXA1 displays very ubiquitous expression in all investigated benign tissues, suggesting a critical role of ANXA1 in the maintenance of gastrointestinal organized tissues. Although ANXA1 expression seems quite unique among healthy human tissues, each tissue shows rather restrictive expression of ANXA1: e.g., highest in the liver, 6.5-fold compared with the calibrator tissue (bile duct). One possible interpretation is that ANXA1 is predominantly localized in the cytoplasm of the epithelial cells, but weak/absent in the stroma. Therefore, the concentration of ANXA1 in certain organ is determined by the ratio of the main cellular components within healthy human tissues.

Although ANXA1 is attracting more attention in cancer research, the conflicting reports on the expression of ANXA1 limited its importance as a therapeutic and/or prognostic biomarker in cancer (Additional file 3: Table S1). Therefore, a systemic investigation with standard methods was necessary to identify the ANXA1 expression profile in six gastrointestinal tumor entities. In the present study, we provided preliminary evidence that ANXA1 expression seems to be tumor type-specific in these malignant tissues. Squamous cell carcinoma of esophagus showed a significant reduction of ANXA1 mRNA and protein expression, which is strongly expressed in the normal squamous epithelium. This finding is compatible with previous studies that identified ANXA1 as a marker of differentiation in squamous cell carcinoma of the cervix, and head and neck [8-10]. Down-regulation of ANXA1 seems to be exceptional in adenocarcinomas, since many clinical cases documented overexpression in adenocarcinoma of various organs, e.g. esophageal and esophagogastric junction, stomach, liver, colon, and pancreas [7,11,13,14,18]. Our results confirmed overexpression of ANXA1 mRNA and protein in pancreatic ductal adenocarcinoma, suggesting ANXA1 up-regulation involved in pancreatic tumorigenesis. However, the trend of ANXA1 up-regulation in tumors was not significant in colorectal and hepatocellular adenocarcinoma, which may be due to limited sample numbers. Interestingly, loss of ANXA1 expression has also been described in some tumors, for example, in breast cancer, cholangiocarcinoma, and gastric cancer [5,21,23]. In present study, however, we observed a significant loss of ANXA1 expression in cholangiocarcinoma and gastric cancer. Taken together, the concept that ANXA1 is “a tumor suppressor” and therefore is usually down-regulated in squamous cell carcinoma can be identified. However, whether ANXA1 is oncogenic in adenocarcinoma requires a new look on the basis of our data.

Using tissue microarray analysis, we previously demonstrated a significant loss of ANXA1 expression in human primary gastric cancer compared with that in adjacent non-neoplastic mucosa. Recently, Cheng et al showed lack of immunoreactivity for AnxA1 in adjacent clinically normal gastric mucosa and overexpression of ANXA1 in GC [18]. Therefore, to clarify the status of ANXA1 expression in gastric mucosa is of great value in determining whether ANXA1 is “oncogenic” or “tumor suppressor”. Firstly, in the immunohistochemistry analysis, we used whole tissue sections of GC to avoid tissue heterogeneity and selected two anti-ANXA1 antibodies after antibody specificity confirmation by Western blotting. Moreover, we applied RT-PCR and immunofluoresncece analysis to evaluate ANXA1 mRNA level and localization. Our study is the first comprehensive study which combined four methods to investigate ANXA1 expression pattern in non-neoplastic mucosa. All these methods uniformly revealed a higher expression of ANXA1 in these gastric epitheliums. Thus, we cannot rule out the possibility of ANXA1 as a tumor suppressor even if ANXA1 staining could not be observed in normal gastric mucosa in those studies. Next, we found that most of the cases investigated showed a lower expression of ANXA1 compared with adjacent non-neoplastic mucosa. In particular, liver metastases showed a lower level of ANXA1 expression than surrounding liver tissues, which serves as an internal control. In line with our previous study, loss of ANXA1 is a frequent event in gastric carcinogenesis. However, upregulation of ANXA1 mRNA was observed in 3 of 8 (37.5%) cases, which might be attributable to an abundant accumulation of cells in the tumor tissue compared with the corresponding benign tissue. Thus, the up-regulation of ANXA1 mRNA would be an epiphenomenon without any functional relevance with carcinogenesis. Taken together, the concept that ANXA1 is “oncogene” and therefore upregulated in GC requires a new looks on the basis of our data.

One of the most important finding of our study is that the level of ANXA1 expression is inversely associated with the cell viability and migration: AGS cells with low ANXA1 expression had higher ability of cell growth and invasion than N87 cells with high ANXA1 expression. By upregulation of ANXA1 with plasmid containing full-length ANXA1, we could significantly inhibit the anchorage-independent cell growth of gastric cancer cells, further supporting the importance of ANXA1 in the progression of gastric cancer.

Mechanically, ANXA1 exerts its antiproliferative activity via the inhibition of cyclin D1 and various signal transducing kinases [2]. Cyclooxygenase-2 (COX-2) is an inducible enzyme and accumulates in activated macrophages and other cells at sites of inflammation. Increasing evidences showed that COX-2 was upregulated in various carcinomas and plays a key role in tumorigenesis. Previously, studies on ANXA1 knockout mice revealed constitutively increased expression of COX-2 [24]. In this context, we found that overexpression of ANXA1 aborgated COX-2 expression in gastric cancer cells. Notably, knockdown ANXA1 expression with ANXA1-specific shRNA leads to an increase of COX-2 expression, suggesting ANXA1 mediating many diverse cellular functions, such as inflammation and proliferation. Moreover, we found that ANXA1 expression inversely co-localized with COX-2 expression in caner specimen, suggesting a cross-talk between ANXA1 and COX-2. This notion is supported by a previous study showing that IL-1beta increased the expression of COX-2 and concomittantly decreased the expression of lipocortin 1 (ANXA1) on the surface of A549 cells [25]. Taken together, our data suggested that the cross-talk between ANXA1 and COX-2 might play a critical role in cell proliferation and tumor growth. However, whether COX-2 is a feedback of cell growth induced by ANXA1 or a direct downstream target of ANXA1 still needs further investigation.

## Conclusions

Collectively, our results provide the systematic survey of ANXA1 for various benign human tissues and tumor specimens. Our data confirmed that ANXA1 expression is different in different types of tumor and plays a multifaceted role in cancer development and progression. Our results also revealed a higher expression of ANXA1 in normal gastric epithelium, suggesting its role in the maintenance of cellular boundaries. Furthermore, ANXA1 inhibits GC cell viability via regulating COX-2 expression. These data revealed important functions of ANXA1 in tumorigenesis and cancer development, but the exact mechanisms still need to be explored.

## Competing interest

There are no competing interests for all authors.

## Authors’ contributions

YG participated in the design of the study, carried out the mRNA and protein expression of ANXA1 in human tissues and cancers and analyzed the data. YC and DX participated the immunohistochemistry analysis of ANXA1 and COX-2 in gastric cancer patients and assisted the analysis of data. YG and DX performed the cell biology study and the Western Blotting test. YC and GY participated evaluation of immunostaining and assisted the collection of clinical data. JW and GY participated in its design and coordination, and supervised the study. GY drafted the manuscript. YG, YC and DX contributed equally to this work. All authors read and approved the final manuscript.

## Pre-publication history

The pre-publication history for this paper can be accessed here:

http://www.biomedcentral.com/1471-2407/14/520/prepub

## Supplementary Material

Additional file 1: Figure S1 Expression profiles of ANXA1 in human well-differentiated tumors and poorly differentiated carcinomas. ESC, esophageal carcinoma; GC, gastric carcinoma; CHO, cholangiocarcinoma. Click here for file

Additional file 2: Figure S2 Expression of ANXA1 in human gastric tissues and cancers by immunohistochemistry using anti-ANXA1 antibody (LS-B3363) obtained from *LifeSpan Biosciences*. Click here for file

Additional file 3: Table S1 Differential expression of ANXA1 in clinical gastrointestinal cancer tissues. Click here for file
