# Supplementary figures and images for: Differential expression of ANXA1 in benign human gastrointestinal tissues and cancers
Source: BMC Cancer. 2014 Jul 19;14:520. doi: 10.1186/1471-2407-14-520 (PMC4223377; doi:10.1186/1471-2407-14-520)

## Slide 1
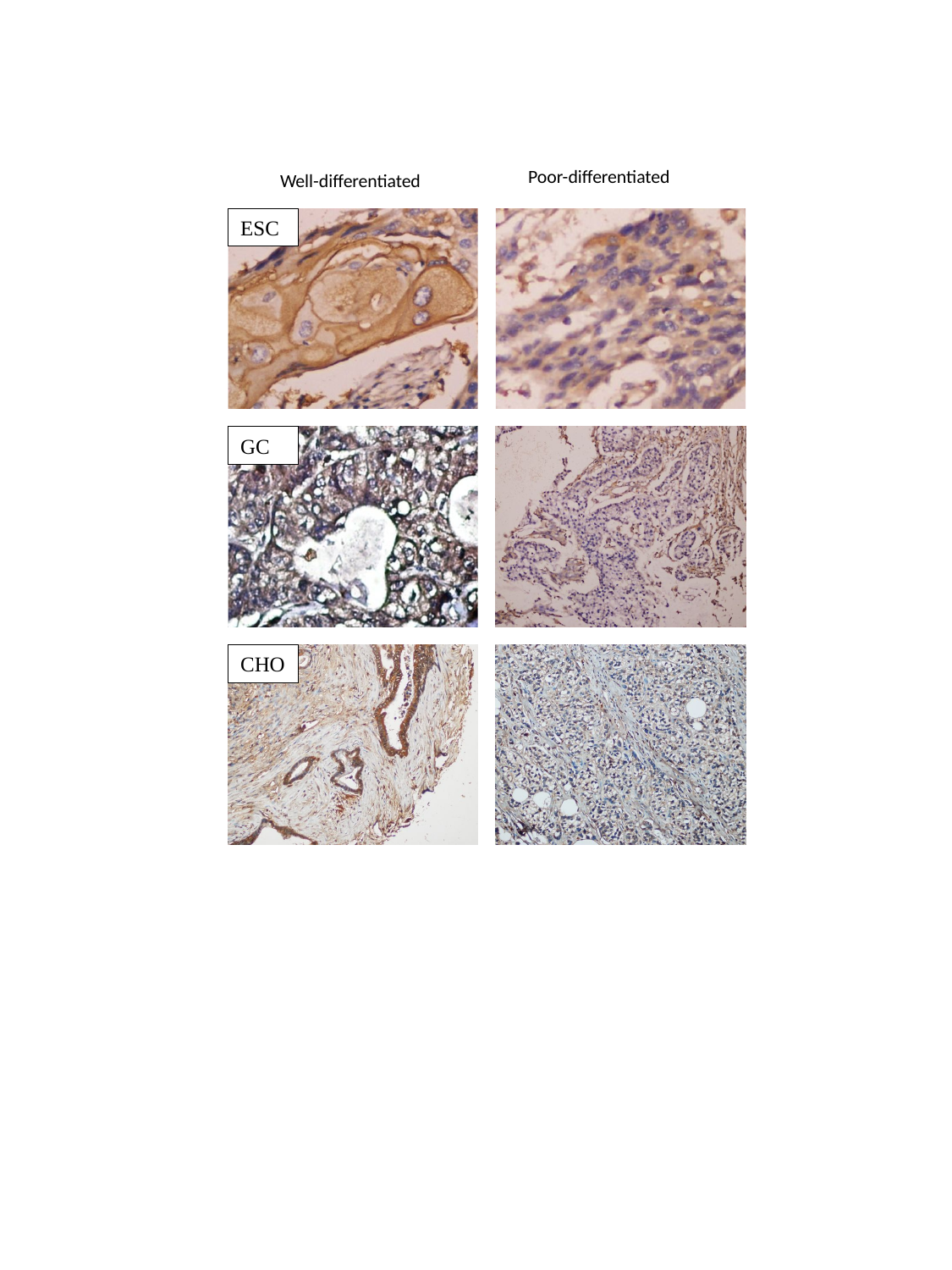

Poor-differentiated
Well-differentiated
ESC
GC
CHO

Supplement: Additional file 1: Figure S1 — Expression profiles of ANXA1 in human well-differentiated tumors and poorly differentiated carcinomas. ESC, esophageal carcinoma; GC, gastric carcinoma; CHO, cholangiocarcinoma. [file 1471-2407-14-520-S1.pptx]

## Slide 1
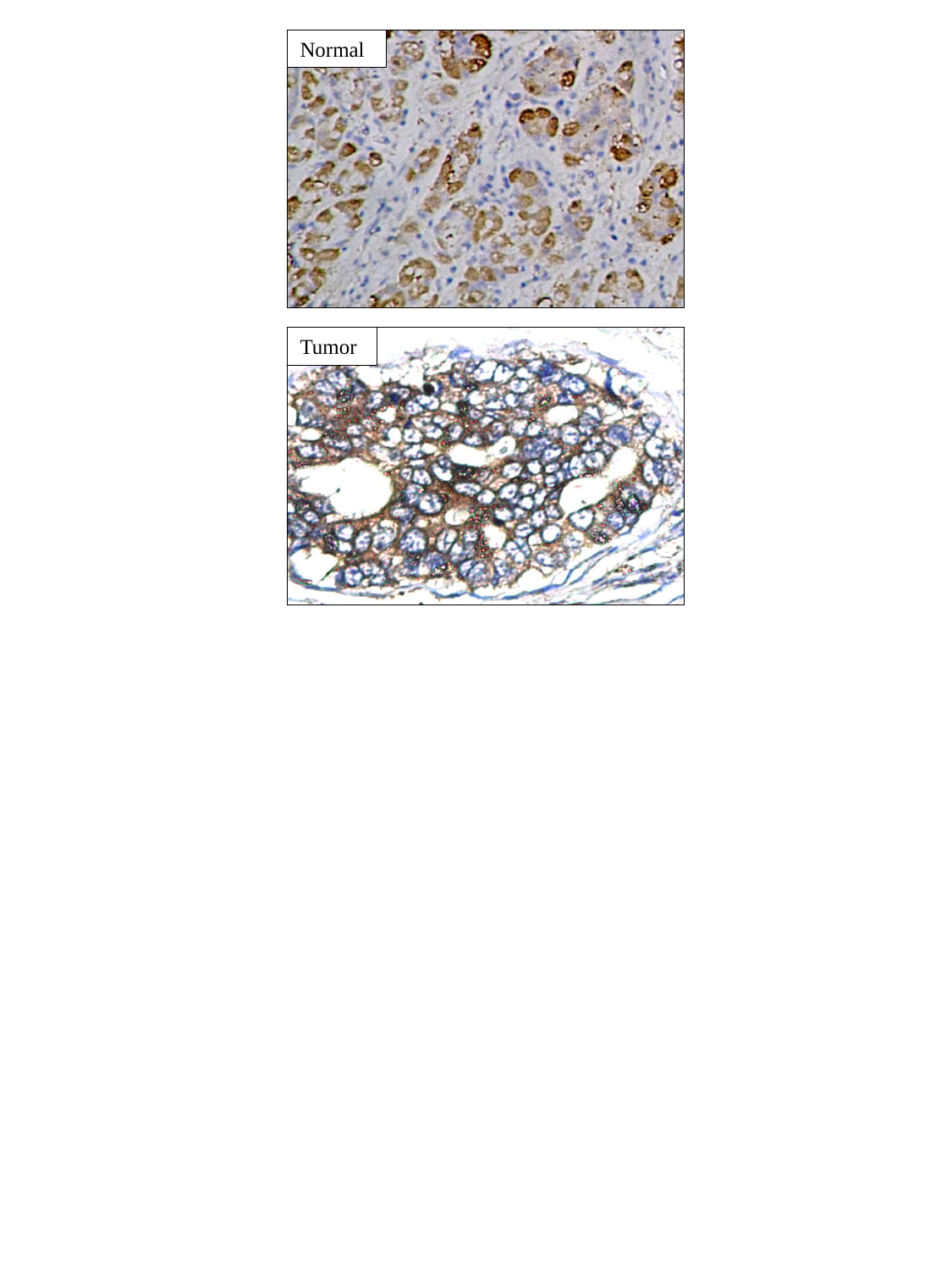

Normal
Tumor

Supplement: Additional file 2: Figure S2 — Expression of ANXA1 in human gastric tissues and cancers by immunohistochemistry using anti-ANXA1 antibody (LS-B3363) obtained from LifeSpan Biosciences. [file 1471-2407-14-520-S2.pptx]
