# Supplementary material for: Differential expression of ANXA1 in benign human gastrointestinal tissues and cancers
Source: BMC Cancer. 2014 Jul 19;14:520. doi: 10.1186/1471-2407-14-520 (PMC4223377; doi:10.1186/1471-2407-14-520)
Supplement: Additional file 3: Table S1 — Differential expression of ANXA1 in clinical gastrointestinal cancer tissues. [file 1471-2407-14-520-S3.docx]

| Tissue | Expression | Normal tissue | Tumor | Tumor type | PMID |
| --- | --- | --- | --- | --- | --- |
|  |  |  | (+/total) |  |  |
| Esophageal | Loss | + | 25/25 | Squamous cell carcinoma | 11103786 |
|  | Loss | + | 17/24 | Squamous cell carcinoma | 12242662 |
|  | Loss | + | 12/16 | Well-differentiated | 12242663 |
|  | Loss | + | 16/17 | Moderately differentiated | 12242664 |
|  | Loss | + | 3/3 | Poorly differentiated | 12242665 |
|  | Loss | + | 30/37 | Squamous cell cancer | 15447985 |
|  | Loss | + | n/a | Squamous cell cancinoma | 17884789 |
|  | Loss | + | n/a | Squamous cell carcinoma | 22441127 |
| Esophageal and Esophagogastric Junction | Increased | +/-* | 41/104 | Adenocarcinoma | 16899607 |
| Gastric | Loss | na | n/a | Sirrhous gastric cell line | 11221876 |
|  | Loss | + | 381/1073 | Adenocarcinoma | 18535914 |
|  | Loss in cytoplasm | + | 47/104 | Adenocarcinoma | 20665809 |
|  | Increased in nuclear | - | 12/104 | Adenocarcinoma | 20665810 |
|  | Increased | - | 76/135 | Adenocarcinoma | 22977491 |
|  | Increased | - | 42/118 | Adenocarcinoma | 22736399 |
| Colorectal | Increased | na | n/a | Adenocarcinoma | 18071363 |
|  | Increased | - | 61/210 | Adenocarcinoma | 22977491 |
| Liver | Increased | + | 31/31 | Hepatocellular carcinoma | 8707286 |
| Bile duct | Loss | + | 27/61 | Hilar cholangiocarcinoma | 20924191 |
| Pancreatic | Increased | +(7/38) | 30/42 | Carcinoma | 15133856 |
|  | Increased | +(7/38) | 24/32 | Ductal adenocarcinoma | 15133856 |

Additional file 3: Table S1. Differential expression of ANXA1 in clinical gastrointestinal cancer tissues

*Esophageal squamous mucosa(+) gastric epithelium(-)
